# Differences in intra-tumoral macrophage infiltration and radiotherapy response among intrinsic subtypes in pT1-T2 breast cancers treated with breast-conserving surgery

**DOI:** 10.1007/s00428-019-02563-3

**Published:** 2019-03-26

**Authors:** Stina Garvin, Eva Vikhe Patil, Lars-Gunnar Arnesson, Husam Oda, Elham Hedayati, Annelie Lindström, Ivan Shabo

**Affiliations:** 1 Division of Pathology, Department of Clinical and Experimental Medicine, Faculty of Medicine and Health Sciences, Linköping University, SE 581 85 Linköping, Sweden; 2 Division of Surgery, Department of Clinical and Experimental Medicine, Faculty of Medicine and Health Sciences, Linköping University, SE 581 85 Linköping, Sweden; 3 Department of Medical Biosciences, Pathology, Umeå University, SE-901 87 Umeå, Sweden; 4 Department of Oncology-Pathology, Karolinska Institutet, SE 171 76 Stockholm, Sweden; 5 Patient Area of Breast Cancer Sarcoma and Endocrine Tumors, Theme Cancer, Karolinska University Hospital, SE 171 76 Stockholm, Sweden; 6 Division of Cell Biology, Department of Clinical and Experimental Medicine, Faculty of Medicine and Health Sciences, Linköping University, SE 581 85 Linköping, Sweden; 7 Endocrine and Sarcoma Surgery Unit, Department of Molecular Medicine and Surgery, Karolinska Institutet, SE 171 77 Stockholm, Sweden

**Keywords:** Breast cancer, Macrophage, CD163, Ki-67, Radiotherapy, Intrinsic subtypes

## Abstract

Breast cancer (BC) intrinsic subtype classification is based on the expression of estrogen receptor (ER), progesterone receptor (PR), human epidermal growth factor receptor 2 (HER2), and proliferation marker Ki-67. The expression of these markers depends on both the genetic background of the cancer cells and the surrounding tumor microenvironment. In this study, we explore macrophage traits in cancer cells and intra-tumoral M2-macrophage infiltration (MI) in relation to intrinsic subtypes in non-metastatic invasive BC treated with breast conserving surgery, with and without postoperative radiotherapy (RT). Immunostaining of M2-macrophage-specific antigen CD163 in cancer cells and MI were evaluated, together with ER, PR, HER2, and Ki-67-expression in cancer cells. The tumors were classified into intrinsic subtypes according to the ESMO guidelines. The immunostaining of these markers, MI, and clinical data were analyzed in relation to ipsilateral local recurrence (ILR) as well as recurrence-free (RFS) and disease-free specific (DFS) survival. BC intrinsic subtypes are associated with T-stage, Nottingham Histologic Grade (NHG), and MI. Macrophage phenotype in cancer cells is significantly associated with NHG3-tumors. Significant differences in macrophage infiltration were observed among the intrinsic subtypes of pT1-T2 stage BC. Shorter RFS was observed in luminal B HER2neg tumors after RT, suggesting that this phenotype may be more resistant to irradiation. Ki-67-expression was significantly higher in NHG3 and CD163-positive tumors, as well as those with moderate and high MI. Cancer cell ER expression is inversely related to MI and thus might affect the clinical staging and assessment of BC.

**Electronic supplementary material:**

The online version of this article (10.1007/s00428-019-02563-3) contains supplementary material, which is available to authorized users.

## Introduction

Breast cancer (BC) is the most common cancer in women worldwide and is composed of multiple subtypes reflecting biologically and pathologically different entities [1, 2]. Surgery of BC entails complete removal of the primary tumor in the breast and a surgical lymph node staging (N-stage) of the axilla. Breast conserving surgery (BCS) in combination with postoperative radiotherapy (RT) is an established treatment of BC and results in equivalent disease-free and overall survival rates compared to mastectomy [3]. The purpose of RT is to eliminate microscopic tumor foci in the conserved breast [4]. After BCS, ipsilateral local recurrence (ILR) of the primary tumor occurs in approximately 10% of breast cancer patients and is associated with increased risk of distant metastases and poor survival [5, 6].

BC classification is based on its underlying biology. The expression of estrogen receptor (ER), progesterone receptor (PR), and human epidermal growth factor receptor 2 (HER2) together with clinicopathological data such as Ki-67-expression, tumor size, tumor grade, and lymph node stage (N-stage), are conventionally used for patient management. Genetically, BC is classified into intrinsic subtypes with distinct clinical outcomes, i.e., luminal A, luminal B, HER2-overexpression, and triple negative (basal-like) tumors. Each of these subtypes can be mapped to an immunohistochemically (IHC) defined phenotype which in turn reflects the biology associated with tumor growth [7–9]. ER/PR and HER2 pathways are involved in cell growth and regulation of cell proliferation. For example, estrogen is a potent breast mitogen and ER inhibitors and estrogen-producing enzymes (aromatases) are well-established, effective BC therapies [10].

Tumor-associated macrophages (TAMs) are a major component of solid tumors [11]. M2-macrophage traits in tumor cells, such as CD163-expression, and intra-tumoral macrophage infiltration (MI) in BC are associated with early tumor recurrence and a poor prognosis [12–15]. Macrophage phenotype in cancer cells is suggested to be caused by fusion between TAMs and cancer cells, which yields hybrid cancer cells with macrophage phenotype [16–18]. Both in vitro and in vivo experimental data support these observations and suggest that cell fusion may play a significant role in tumor progression [17]. Moreover, cell fusion may contribute to tumor heterogeneity by creating subsets of tumor cells with reduced susceptibility to chemo- and radiotherapy [15, 19–21]. The expression of macrophage traits by cancer cells is associated with MI suggesting that increased recruitment of macrophages in tumor tissue might result in higher rates of fusion between macrophages and cancer cells in tumor stroma [15, 22]. Moreover, experimental studies have shown that TAMs contribute to increased tumor cell proliferation and induce decreased cancer cell expression of ER and PR in BC [23, 24]. Thus, there appears to be an interaction between TAMs, BC phenotype, and thereby clinical histopathological assessment and treatment. Little is known, however, regarding intra-tumoral density of TAMs and macrophage phenotype in cancer cells in relation to patient prognosis, tumor differentiation, tumor proliferation, and intrinsic subtype in BC.

In this study, we use a unique clinical material consisting of patients with non-metastatic invasive BC treated with breast conserving surgery, with and without postoperative radiotherapy (RT) in order to examine intrinsic subtypes with regard to macrophage phenotype, MI, and radiotherapy response in terms of ILR, recurrence-free survival and disease-free survival.

## Materials and methods

### Patient material and study design

**Fig. 1:**
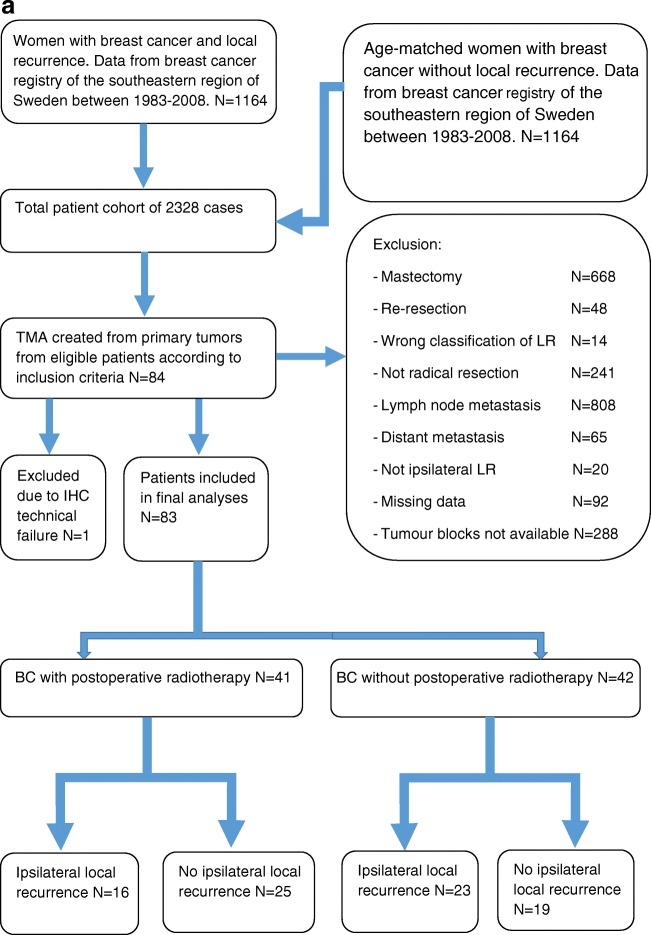

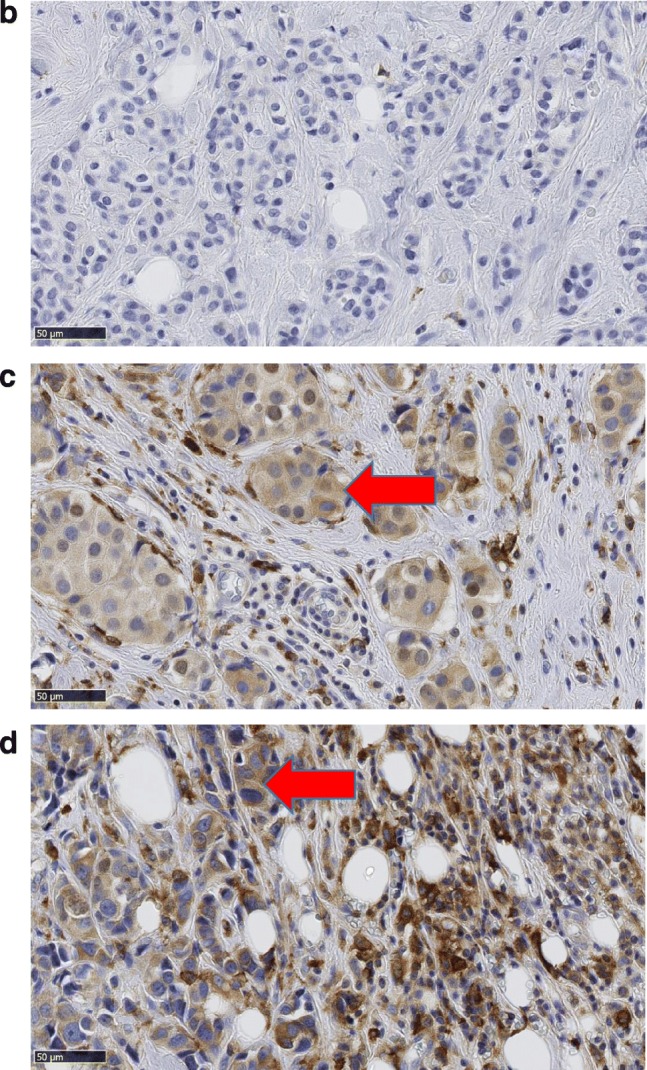
**a** Flow diagram showing the selection of 83 breast cancer patients treated with breast conserving surgery. All patients had no lymph node or distant metastasis at the time of surgery and the tumors were completely removed. **b**–**d** Immunohistochemistry for CD163 used for scoring of macrophage infiltration and evaluation of CD163-expression in breast cancer cells. In **b**, low macrophage infiltration and breast cancer cells negative for CD163. In **c**, moderate macrophage infiltration and breast cancer cells positive for CD163 (red arrow). In **d**, high macrophage infiltration and some breast cancer cells positive for CD163 (red arrow)

We collected data on all patients (*n* = 1164) with BC with isolated ipsilateral local recurrence (ILR) during the years of 1983–2008 from the breast cancer registry of the southeastern region of Sweden. For comparison, we selected an age-matched patient cohort (*n* = 1164), treated during the same period and without ILR. Only patients with radically removed tumors (R0), without lymph node metastases (N0) or distant metastases (M0) were included. All patients were treated with conventional BCS at surgical departments within the county of Östergötland, Sweden. Using this retrospective design, we were able to include patients who were not offered RT, as it was not fully implemented in clinical routine until the early 1990s [25], thus allowing for investigations into possible associations between intrinsic subtypes, Ki-67 expression, and tumor recurrence in relation to RT (Fig. 1a). Ethical approval from the Regional Ethics Committee in Linköping was obtained according to Swedish Biobank Law (reference number: 2010/311–31).

Tumor histology was reviewed by an experienced pathologist (SG), and formalin-fixed paraffin-embedded tissue blocks with invasive BC were chosen for tissue microarray, constructed using two tissue cores (diameter 0.6 mm). Eighty-three patient samples were included in total. Liver samples were used as a position control.

### Immunostaining and antibodies

CD163 is considered to be a macrophage-specific marker and is generally not expressed in cell types other than monocytes/macrophages [26]. Based on the cell fusion theory, we used CD163-expression as a surrogate marker for macrophage phenotype in breast cancer cells [16, 27]. Five-micrometer sections were obtained from formalin-fixed paraffin-embedded TMA tumor specimens. The sections were de-paraffinized in xylene and hydrated in a series of graded alcohols, pretreated with heat-induced epitope retrieval and trisethylenediaminetetraacetic acid buffer (1 mM, pH 9, 20/5/20 min; Decloaking Chamber NxGen, Biocare Medical), and stained for CD163 (anti-human, monoclonal antibody, clone 10D6, Novocastra, Leica). Staining for estrogen receptor (ER; clone SP1, Ventana Roche), progesterone receptor (PR; clone 1E2, Ventana Roche), Ki-67 (clone MIB-1, Dako Agilent), and human epidermal growth factor receptor 2 (HER2; clone 4B5, Ventana Roche) was done according to clinical laboratory standards. All slides were scanned to digital images using the Hamamtsu NanoZoomer XL (Visiopharm LRI AB). Evaluation of immunostaining was performed using ImageScope viewing software (Leica Biosystems).

### Evaluation of immunostaining

All immunostaining was evaluated by two experienced pathologists (SG and HO), blinded to patient characteristics and outcome. Macrophages and cancer cells were distinguished histomorphologically, the macrophages exhibiting small, regular nuclei and the cancer cells atypical nuclei with variations in size, shape, and chromatin staining. TAM infiltration was evaluated semi-quantitatively, classified in three categories: no/low, moderate, or high [22, 28] (Fig. 1b–d). The fraction of CD163-positive cancer cells was calculated based on a count of 200 tumor cells in each TMA core. The tumors were considered CD163-positive if > 15% of the tumor cells expressed CD163 [15]. The expression of Ki-67, ER, PR, and HER2 in cancer cells was evaluated according to ESMO guidelines (2017) [29].

### Intrinsic subtype classification

All tumors were assigned an intrinsic molecular subtype of breast cancer (luminal A, luminal B, HER2-overexpression, and triple negative) according to ESMO guidelines (2017) [29, 30]. The criteria for intrinsic classification are summarized in Table 1, supplement data.

### Statistical analysis

SPSS statistics software, version 25 (IBM Corporation, USA), was used for the statistical analyses. CD163-expression and MI were evaluated in relation to intrinsic subtypes, NHG, and clinicopathologic data using Pearson’s chi-square test. One-way analysis of variance (ANOVA) was used together with a post hoc Bonferroni’s test for analysis of continuous data. Survival rates were estimated according to Kaplan-Meier based on recurrence-free survival (RFS) and disease-free survival (DFS). The statistical significance of differences between survival rates was determined by the log-rank test. For all analyses, *p* < 0.05 was considered statistically significant.

## Results

Out of 83 patients included in the study, 41 (49%) patients received RT after BCS. ILR occurred in 39 (47%) patients, of which 16 (41%) had received RT. Patient characteristics and clinicopathological data are summarized in Table 1.

**Table 1:** Patient characteristics

Variables	*N* (%)
Age groups (years)	
≤ 40	15 (18)
41–50	18 (22)
51–60	17 (20)
61–70	15 (18)
≥ 70	18 (22)
Pathologic T-stage	
pT1a	4 (5)
pT1b	23 (28)
pT1c	43 (51)
pT2	13 (16)
Nottingham grading system	
NHG 1	20 (24)
NHG 2	38 (46)
NHG 3	25 (30)
ER status	
Negative	14 (17)
Positive	66 (79)
Missing data	3 (4)
PR status	
Negative	23 (28)
Positive	58 (70)
Missing data	2 (2)
HER2 status	
Negative	73 (88)
Positive	6 (7)
Missing data	4 (5)
Proliferation index	
Ki-67 < 14%	46 (55)
Ki-67 ≥ 14%	32 (39)
Missing data	5 (6)
Postoperative radiotherapy	
No	42 (51)
Yes	41 (49)
Local recurrence	
No	44 (53)
Yes	39 (47)
CD163-expression in breast cancer cells	
Negative (< 15%)	64 (77)
Positive (≥ 15%)	17 (21)
Missing data	2 (2)
Macrophage infiltration	
No/low	41 (49)
Moderate	28 (34)
High	12 (15)
Missing data	2 (2)

### Intrinsic subtypes

The distribution of intrinsic subtypes was as follows: luminal A 43 (52%), luminal B HER2-positive (HER2pos) 5 (6%), luminal B HER2-negative (HER2neg) 14 (17%), and triple negative 11 (13%) patients. There were no cases in the HER2-overexpression subtype.

Significant differences were found in the distribution of pathologic tumor stage (T-stage) (*p* = 0.04), NHG (*p* = 0.002), and MI (*p* = 0.001) in relation to intrinsic subtypes. pT2 stage was more common in triple-negative tumors (45%, 5 of 11) compared to luminal A (12%, 5 of 43), luminal B HER2pos (0%, 0 of 5), and luminal B HER2neg (21%, 3 of 14; Table 2).

**Table 2:** Univariate analysis examining age, pathologic T-stage, Nottingham grade, intra-tumoral macrophage infiltration, and CD163-expression by tumor cells among the intrinsic subtypes of breast cancer

Intrinsic subtypes					
	Luminal A N (%)	Luminal B HER2pos N (%)	Luminal B HER2neg N (%)	Triple negative N (%)	*P*
Age groups (years)					
≤ 40	5 (12)	1 (20)	4 (29)	2 (18.2)	
41–50	8 (18)	2 (40)	3 (21)	3 (27.3)	
51–60	11 (26)	1 (20)	2 (14.3)	1 (9.1)	
61–70	9 (21)	1 (20)	2 (14.3)	1 (9.1)	
≥ 70	10 (23)	0 (0)	3 (21.4)	4 (36.3)	0.83
Pathologic T-stage					
pT1	38 (88)	5 (100)	11 (79)	6 (54.5)	
pT2	5 (12)	0 (0)	3 (21)	5 (45.5)	0.04
Nottingham grading system					
NHG 1	14 (33)	0 (0)	1 (7)	0 (0)	
NHG 2	23 (53)	3 (60)	6 (43)	3 (27)	
NHG 3	6 (14)	2 (40)	7 (50)	8 (73)	0.002
CD163-expression					
Negative (< 15%)	37 (86)	2 (50)	10 (71)	8 (73)	
Positive (≥ 15%)	6 (14)	2 (50)	4 (29)	3 (27)	0.25
Macrophage infiltration					
Low	31 (72)	1 (25)	3 (21)	2 (18)	
Moderate	10 (23)	3 (75)	3 (43)	5 (46)	
High	2 (5)	0 (0)	5 (36)	4 (36)	0.001

Luminal A phenotype was less commonly represented among poorly differentiated tumors (NHG3) (14%, 6 out of 43). The corresponding rates in NHG3 tumors with luminal B HER2pos, luminal B HER2neg, and triple-negative subtypes were 40% (2 of 5), 50% (7 of 14), and 73% (8 of 11), respectively (*p* = 0.002; Table 2).

### Macrophage infiltration

**Fig. 2:**
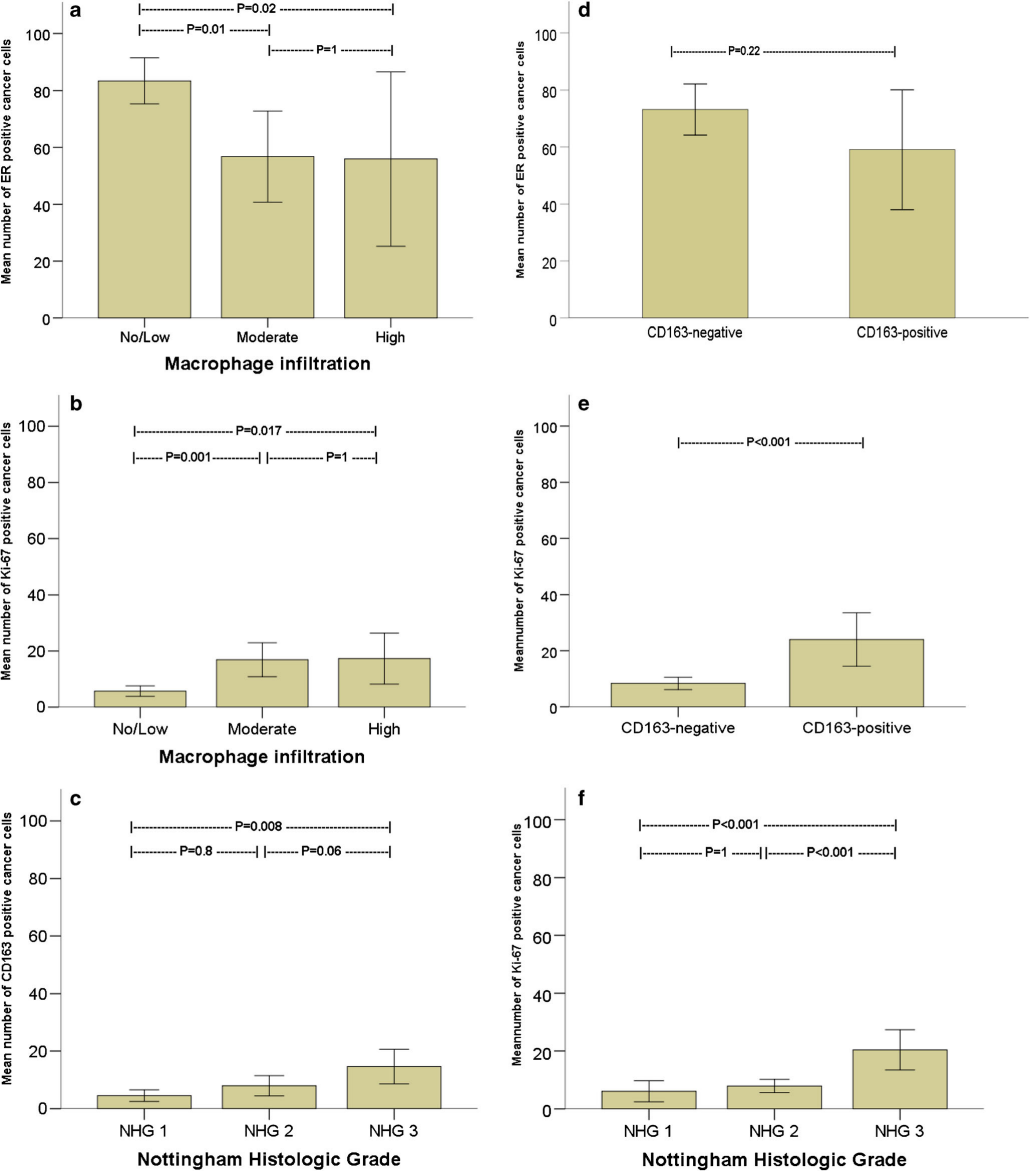
The relation of macrophage infiltration to **a** ER expression (mean ± 95% CI) and **b** Ki-67-expression (mean ± 95% CI) in breast cancer cells. In **c**, comparing the mean number of cancer cells expressing CD163 in relation to NHG. In **d**, estrogen receptor (ER) expression in relation to CD163-positive and CD163-negative breast cancers. The proportion of Ki-67-positive breast cancer cells (mean ± 95% CI) for **e** CD163-positive and CD163-negative breast cancers as well as **f** in relation to Nottingham Histologic Grade (NHG). All comparisons are based on ANOVA followed by Bonferroni’s post hoc test

High MI was more common in luminal B HER2neg tumors (36%) and triple-negative tumors (36%) compared to luminal A (5%) and luminal B HER2pos (0%) subtypes (*p* = 0.001). Of the 43 luminal A subtype cases, 31 (72%) were classified as low MI, 10 (23%) as moderate MI, and 2 (5%) as high MI (Table 2). Since the luminal A subtype is mainly based on ER expression, we further examined MI in relation to ER expression in cancer cells. ER expression in cancer cells was significantly higher in tumors with low MI compared to moderate (*p* = 0.01) and high MI (*p* = 0.02; Fig. 2a). Conversely, the proliferative index measured by Ki-67-expression was significantly lower in tumors with low MI compared to moderate (*p* = 0.001) and high MI (*p* = 0.017; Fig. 2b). Ki-67-expression was similar in tumors with moderate and high MI.

### CD163-expression in breast cancer cells

CD163 expression (of any proportion) in breast cancer cells was found in 43 patients (53%). In two cases, CD163 expression could not be evaluated due to technical failure. The mean proportion of CD163-positive cells in all tumors was 9% (range 0–41%). CD163 expression > 15% was chosen as the cut-off for defining CD163-positivity in further analyses [15]. Applying this cut-off, 17 of 81 (21%) tumors were defined as CD163-positive (Table 1).

The proportion of cancer cells expressing CD163 was higher in NHG3 tumors compared to NHG1 (*p* = 0.008) and NHG2 tumors (*p* = 0.06; Fig. 2c). No significant differences were found in CD163 expression according to intrinsic subtypes (Table 2). ER expression in cancer cells appeared higher in CD163-negative tumors compared to CD163-positive tumors, but this difference was not statistically significant (*p* = 0.22; Fig. 2d). Ki-67-expression was significantly higher in CD163-positive tumors (*p* < 0.001; Fig. 2e).

### Intrinsic subtypes, Ki-67-expression, radiotherapy, and tumor recurrence

As expected, Ki-67-expression was significantly higher in NHG3 tumors compared to NHG1 (*p* = 0.001) and NHG2 tumors (*p* < 0.001). NHG1 and NHG2 tumors showed no significant difference in Ki-67 expression (Fig. 2f).

**Fig. 3:**
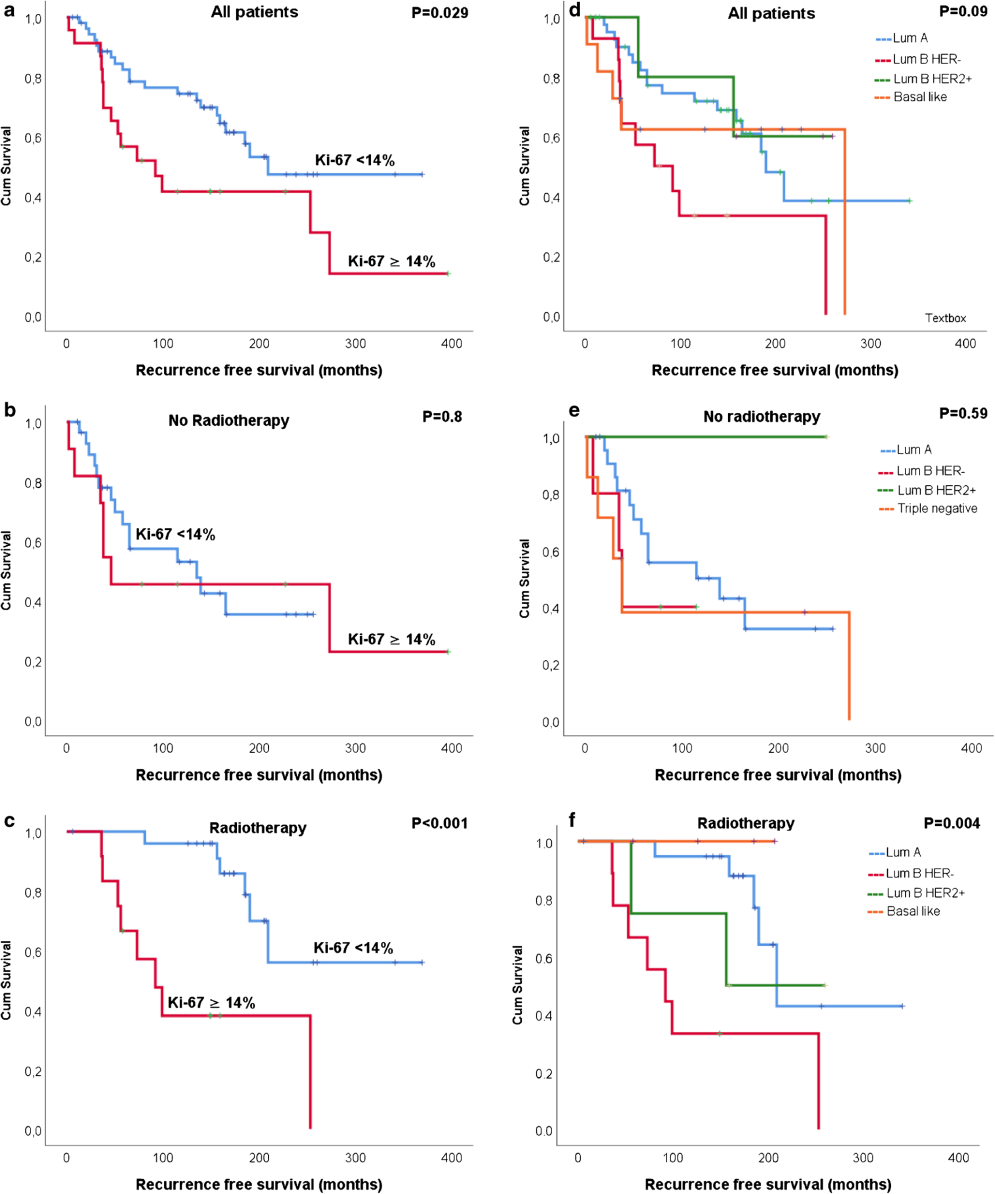
Kaplan-Meier curves demonstrating recurrence-free survival in relation to **a**–**c** proliferation index and **d**–**e** intrinsic subtypes for 83 patients with non-metastasized pT1-pT2 breast cancers treated with breast conserving surgery. The survival analysis is based on time to ipsilateral local recurrence rates and comparison is estimated according to log-rank (Mantel–Cox) test

Regardless of RT, ILR occurred significantly earlier in patients with high proliferative tumors (Ki-67 ≥ 14%) compared to patients with low proliferative tumors (156 months compared to 234 months; *p* = 0.029) (Table 3). In patients who did not receive RT, no significant difference in RFS was found between patients with high and low proliferative tumors (167 and 138 months respectively; *p* = 0.8). After RT, patients with low proliferative tumors had significantly longer RFS compared to those with high proliferative tumors (285 compared to 137 months; *p* < 0.001; Fig. 3a–c).

**Table 3:** Univariate analysis examining ipsilateral local recurrence after breast conserving surgery in relation to radiotherapy, age, intrinsic subtypes, proliferation index, Nottingham Histologic Grade (NHG), CD163-expression in tumor cells, and macrophage infiltration in breast cancer

	No radiotherapy			Radiotherapy		
	Local recurrence			Local recurrence		
	No N (%)	Yes N (%)	*P*	No N (%)	Yes N (%)	*P*
Age group						
≤ 40	2 (10)	6 (26)		1 (4)	6 (37)	
41–50	1 (5)	5 (22)		6 (24)	6 (38)	
51–60	6 (32)	3 (13)		7 (28)	1 (6)	
61–70	4 (21)	1 (4)		8 (32)	2 (13)	
≥ 70	6 (32)	8 (35)	0.11	3 (12)	1 (6)	0.024
Intrinsic subtypes Ki-67						
Luminal A	11 (69)	12 (60)		15 (65)	5 (36)	
Luminal B HER2pos	1 (6)	0 (0)		2 (9)	2 (14)	
Luminal B HER2neg	2 (12.5)	3 (15)		2 (9)	7 (50)	
Triple negative	2 (12.5)	5 (25)	0.55	4 (17)	0 (0)	0.017
Proliferation index						
Ki-67 < 14%	14 (78)	15 (68)		20 (83)	6 (43)	
Ki-67 ≥ 14%	4 (22)	7 (32)	0.5	4 (17)	8 (57)	0.01
Nottingham grading system						
NHG1	4 (21)	5 (22)		8 (32)	3 (19)	
NHG2	9 (47)	10 (43)		12 (48)	7 (44)	
NHG3	6 (32)	8 (35)	1	5 (20)	6 (37)	0.5
CD163 expression						
Negative (< 15%)	15 (79)	16 (73)		21 (84)	3 (20)	
Positive (≥ 15%)	4 (21)	6 (27)	0.64	4 (16)	3 (20)	0.75
Macrophage infiltration						
Low	11 (58)	11 (50)		12 (48)	7 (47)	
Moderate	5 (26)	8 (36)		10 (40)	5 (33)	
High	3 (16)	3 (14)	0.8	10 (3)	3 (20)	0.8

In patients who did not receive RT, there were no associations observed between RFS and intrinsic subtypes (*p* = 0.59). On the other hand, in patients who received RT, RFS was shorter for those with luminal B HER2neg tumors compared to the other intrinsic subtypes (*p* = 0.004; Fig. 3d–f). For DFS, no associations were found with Ki-67-expression or with intrinsic subtype, regardless of RT (supplement data).

## Discussion

Both intrinsic properties of breast cancer cells and TAMs are involved in tumor progression [31, 32]. In this study, we investigated associations between intrinsic subtypes in relation to MI, the expression of macrophage traits in cancer cells, and response to RT in a well-defined patient cohort treated with BCS, with and without postoperative RT. Here, we show that BC intrinsic subtypes are associated with T-stage, NHG, and MI. ER expression is inversely related to intra-tumoral macrophage density. In the group who did not receive radiotherapy, no statistical differences were observed in RFS in relation to intrinsic subtypes. However, in the group that received radiotherapy, the patients with luminal B HER2neg tumors showed a significantly shorter relapse-free survival.

NHG3 tumors showed a significantly higher proportion of CD163-expressing tumor cells, suggesting an association between macrophage phenotype and poor differentiation. Not surprisingly, high proliferative index was observed in NHG3 tumors, and high proliferative index was also observed in CD163-positive tumors. Tumors with moderate and high MI also showed a significantly lower ER expression and higher proliferation index.

### Intrinsic subtypes as a biological concept explaining ILR

When the intrinsic subtype classification was reported almost 18 years ago by Sørlie et al. and Perou et al. [7, 8], postoperative RT was a routine treatment after BCS and thus, this classification has not been explored in a clinical material with patients who have not been treated with radiation [33]. To our knowledge, this is the first study where BC intrinsic subtypes are investigated in relation to ILR and disease-free survival after BCS treated with and without RT.

After BCS, luminal A cancers relapse two to three times less often than HER2-positive and triple negative lesions [34]. In this study, when looking at the group of patients who received radiotherapy, we observed significantly shorter recurrence-free survival in patients with luminal B HER2neg tumors compared to the other subtypes [35]. Differences in recurrence-free survival according to subtype were not observed in the group of patients who had not received radiotherapy, suggesting that the effect of RT may be influenced by the tumor’s intrinsic subtype. These findings, in line with those reported in previous studies [36], reinforce the concept that the prognosis of ILR of BC is mainly driven by the biology of the disease.

### Macrophage infiltration and breast cancer classification

TAMs contribute to tumor progression by promoting angiogenesis, matrix remodeling, tumor cell proliferation, immune evasion, invasion, and metastasis [37, 38]. In vivo macrophage depletion delays the progression of preinvasive lesions into metastatic carcinomas and inhibits development of secondary tumors while early recruitment of macrophages in tumor stroma accelerates tumor progression and invasion [39, 40]. Consistent with these experimental data, clinical studies have shown that increased MI in BC is associated with advanced tumors and poor prognosis [41].

Moreover, macrophages are involved in downregulation of ER expression in breast cancer cells by paracrine-mediated transcriptional repression, independent of the intrinsic phenotype of the cancer cells themselves [23]. Consistent with these in vitro data, this study shows that MI is inversely related to cancer cell ER expression. Moreover, the distribution of the intrinsic subtypes differed significantly among tumors with low, moderate, and high MI.

These observations are likely to have clinical relevance as downregulation of ER in breast cancer cells will change the pathologic staging and the treatment options for the patients. Taking to consideration that the influence of TAMs may vary within the tumor (spatially, contributing to tumor heterogeneity) and during different stages of tumor progression (temporally), macrophage-induced downregulation of ER may potentially influence clinical response to endocrine treatment [42].

### Macrophage phenotype in cancer cells and breast cancer classification

CD163 is a macrophage-specific transmembrane scavenger receptor and a marker of the M2-macrophage phenotype [43]. Macrophage traits in cancer cells, defined by CD163-expression, have been reported for several tumor types, among others renal cell carcinoma [44], breast [21], colorectal [13], and bladder cancers. Moreover, breast cancer cells also express other macrophage markers such as MAC387 [12], DAP12 [45], and CD45 [21]. Accumulating in vitro [16, 46], in vivo [17, 18], and clinical evidence [27, 47] indicate that macrophage traits in cancer cells result from fusion between TAMs and cancer cells. This fusion process yields hybrid cells that acquire genetic and phenotypic characteristics from both maternal cells and that exhibit a metastatic phenotype [27]. Moreover, the macrophage-breast cancer cell hybrids acquire stem cells properties [48] and display morphologic and genetic heterogeneity [21].

In our earlier studies, we have demonstrated that CD163 expression in breast cancer cells is associated with advanced tumor stage [12] and shorter disease-free survival but we have not observed an association with ILR [15]. In this study, we observed a significantly higher proportion of CD163-expressing tumor cells in NHG3-tumors, but we observed no differences in CD163 expression among intrinsic subtypes.

The rationale behind the intrinsic subtypes is that breast cancers are clustered into groups differentiated by expression patterns of co-expressed gene clusters related mainly to proliferation, hormone receptor signaling (luminal cluster), HER2 signaling, and basal epithelial cells of the breast. These gene panels and their immunohistochemically corresponding groups are mainly related to tumor growth [7, 8]. Cell fusion yields new cell clones that are genetically distinct from their maternal cell populations, contributing to tumor heterogeneity by causing transcriptional changes and acquisition of mesenchymal associated phenotypes [18]. Although not statistically significant, we observed a trend suggesting an inverse relationship between ER expression and CD163 expression in BC tumor cells, consistent with findings from previous studies [12, 49]. The observations in this study illustrate the complex relationship between macrophage phenotype, ER expression, and differentiation, and raise the hypothesis that macrophage-breast cancer cell fusion may contribute to genotypic and immunophenotypic features reflected in the intrinsic subtypes.

## Conclusion

Significant differences in macrophage infiltration are observed among the intrinsic subtypes of pT1-T2 stage BC. Shorter recurrence-free survival was observed in luminal B HER2-negative tumors after RT, suggesting that this phenotype may be more resistant to irradiation. Tumor cell expression of ER is inversely related to macrophage infiltration. This study demonstrates new insights into the biological and clinical significance of macrophages in BC.

## Electronic supplementary material

ESM 1 Kaplan-Meier curves demonstrating disease-free survival in relation to (A-C) proliferation index and (D-E) intrinsic subtypes for 83 patients with non-metastasized pT1-pT2 breast cancers treated with breast conserving surgery. The comparison is estimated according to log-rank (Mantel–Cox) test. (DOCX 101 kb)
