# Supplementary material for: Differences in intra-tumoral macrophage infiltration and radiotherapy response among intrinsic subtypes in pT1-T2 breast cancers treated with breast-conserving surgery
Source: Virchows Arch. 2019 Mar 26;475(2):151–62. doi: 10.1007/s00428-019-02563-3 (PMC6647441; doi:10.1007/s00428-019-02563-3)
Supplement: Supplementary file 1 — Kaplan-Meier curves demonstrating disease-free survival in relation to (A-C) proliferation index and (D-E) intrinsic subtypes for 83 patients with non-metastasized pT1-pT2 breast cancers treated with breast conserving surgery. The comparison is estimated according to log-rank (Mantel–Cox) test. (DOCX 101 kb) [file 428_2019_2563_MOESM1_ESM.docx]

**Supplementary data**

**Table 1. Categories of Intrinsic subtypes according to ESMO guidelines (2017).**

| **Intrinsic subtype** | **IHC status** |
| --- | --- |
| Luminal A | ER-positive, PR-positive, HER2-negative and Ki-67<14% |
| Luminal B  HER2-negative | ER-positive, HER2-negative, and either Ki-67 ≥14% or PR-negative |
| Luminal B  HER2-positive | ER-positive, HER2-positive, any Ki-67, any PR |
| HER2-overexpression | HER2-positive, ER-negative, and PR-negative |
| Triple negative | ER-negative, PR-negative, HER2-negative |
| ER, oestrogen receptor; HER2, Human epidermal growth factor receptor 2; PR, progesterone receptor. | |

**Figure 1.**


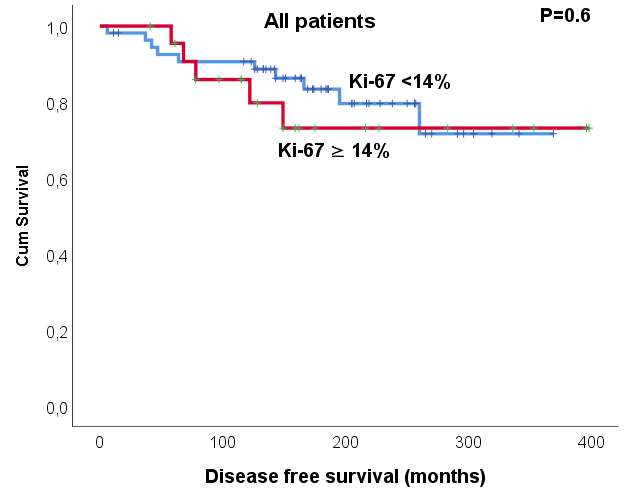

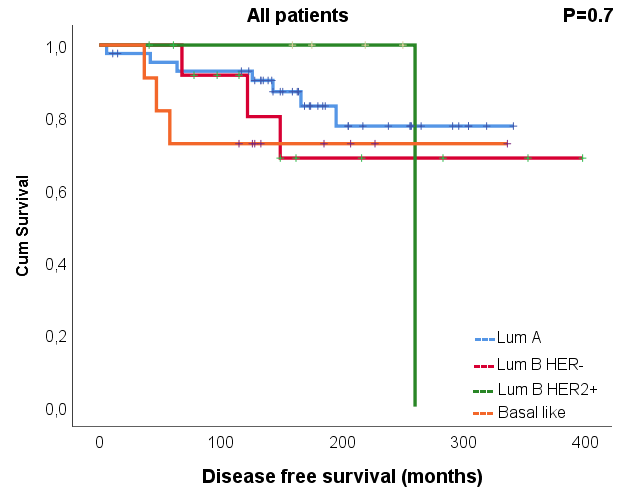


**A D**


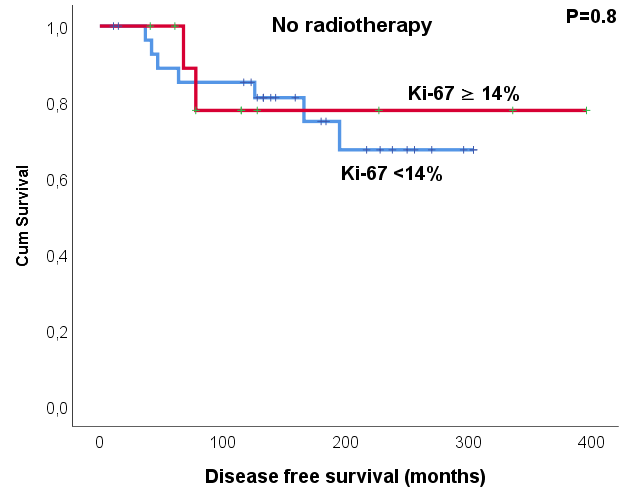

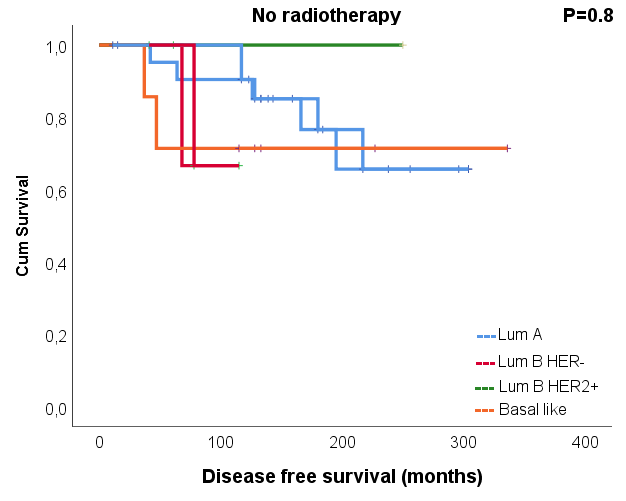


**B E**


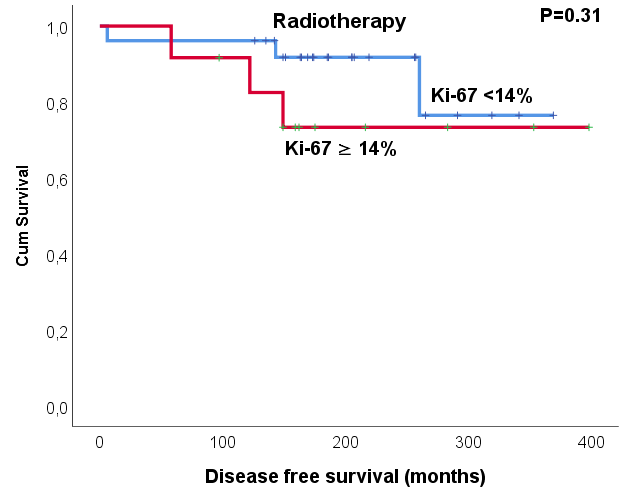

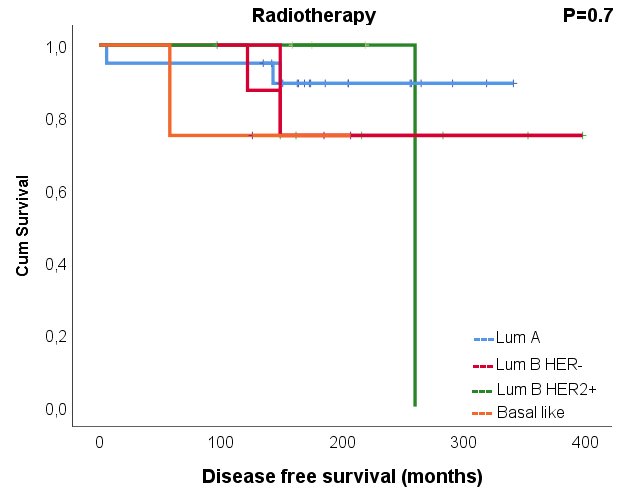


**C F**
